# MicroRNAs Control Macrophage Formation and Activation: The Inflammatory Link between Obesity and Cardiovascular Diseases

**DOI:** 10.3390/cells3030702

**Published:** 2014-07-10

**Authors:** Richard Cheng-An Chang, Wei Ying, Fuller W. Bazer, Beiyan Zhou

**Affiliations:** 1 Department of Veterinary Physiology and Pharmacology, Texas A&M University, College Station, TX 77843, USA; 2 Department of Animal Science, Texas A&M University, College Station, TX 77843, USA

**Keywords:** microRNA, macrophage, obesity, cardiovascular diseases

## Abstract

Activation and recruitment of resident macrophages in tissues in response to physiological stress are crucial regulatory processes in promoting the development of obesity-associated metabolic disorders and cardiovascular diseases. Recent studies have provided compelling evidence that microRNAs play important roles in modulating monocyte formation, macrophage maturation, infiltration into tissues and activation. Macrophage-dependent systemic physiological and tissue-specific responses also involve cell-cell interactions between macrophages and host tissue niche cell components, including other tissue-resident immune cell lineages, adipocytes, vascular smooth muscle and others. In this review, we highlight the roles of microRNAs in regulating the development and function of macrophages in the context of obesity, which could provide insights into the pathogenesis of obesity-related metabolic syndrome and cardiovascular diseases.

## 1. Introduction

Resident immune cells are the critical coordinators for the maintenance of the physiological homeostasis of certain tissues, as well as systemically. Among a wide variety of tissue-resident immune cells, macrophages can account for the major population within the stromal cell population [1]. However, once the balance between resident macrophages and the tissue *per se* within the microenvironment is lost, the aberrant effects from resident macrophages could lead to abnormal tissue functions. For example, chronic low-grade adipose tissue inflammation is associated with obesity, which is accompanied by enhanced immune cell infiltration that is a hallmark of obesity and a crucial contributor to the pathogenesis of insulin resistance and other metabolic diseases [2,3]. Among these immune cells, a significant accumulation of macrophages can account for up to 50% of the stromal cell population in the adipose tissue of obese individuals [4]. On the other hand, during the development of atherosclerotic plaques, the vascular endothelial response to the compromised circulation is the recruitment of circulating monocytes through increased expression of inflammatory adhesion molecules, including the intercellular adhesion molecule (ICAM) [5], the vascular cell adhesion molecule (VCAM) [6] and the chemokine (C-C motif) ligand 2 (CCL2, also known as monocyte chemoattractant protein-1, MCP-1) [7]. Vascular macrophages reside in the specific areas and function as both immune cells and as scavengers to “clean up” the abnormal tissue debris in the plaque. However, both the plaque niche and the nutritional composition of the circulation can affect macrophage morphology and function. After attempting to remove the aggregated lipoproteins within the plaque, as well as taking up circulating cholesterol, the resident macrophages in the vascular endothelium are filled with lipids in the cell body and appear to have a “foamy” morphology; thus, they are named foam cells. Therefore, as a critical tissue-infiltrating cell type, foam cell macrophages have been the target population in the study of plaque formation for a long time. To date, the infiltrated macrophages have been one of the focal points of atherosclerosis research and are believed to be critical in regulating changes in the local environment. 

The importance of microRNAs (miRNAs)is well recognized in regulating genetic networks and subsequent physiological processes. Dysregulated expression levels of miRNAs in human and animal models are associated with various diseases conditions and their progression, including obesity-associated metabolic syndromes and cardiovascular diseases. miRNAs provide a new layer of gene regulation at the post-transcriptional level in controlling gene expression and their involved signaling pathways. By binding to the 3'untranslated region of mRNAs, miRNAs can suppress target protein production by inducing RNA degradation and/or blocking translation. For example, miR-126-5pcan promote vascular repair through targetingDlk1 [8]. Shear stress and hypercholesterolemia induce the expression of miR-92a, which, in turn, elevates endothelial activation, leading to the development of atherosclerosis [9]. However, how miRNAs regulate tissue-resident immune cell function, specifically macrophages, in the disease context is not well understood. 

The recent discovery of extracellular miRNAs opens a new window to understanding the functional mechanisms of this small non-coding RNA family. Interestingly, the expression patterns of extracellular miRNAs are correlated with various diseases, including type 2 diabetes and atherosclerosis, suggesting their potential roles serving as biomarkers for diagnosis. The serum levels of miR-125a-5p, miR-126-3p, miR-221-3p and miR-222-3p are reduced in patients with atherosclerosis compared to healthy subjects [10]. Extracellular miRNAs may present in different formats, including exosomal particles, microvesicles and argonaute-bound molecules. However, major questions regarding their secretion and remote tissue targeting remain unanswered. For example, extracellular miRNAs can be passively released by damaged tissues and cells or be actively secreted by cells, acting as cell-cell communication factors. Further endeavors are necessary to address these questions so that translational strategies can be developed for therapeutic practice. Here, we review current progress about the importance of miRNAs in regulating macrophage maturation and their functions in different tissue types, including adipose tissues and the vascular system, as well as plasma miRNAs.

## 2. miRNA-Regulated Macrophage Maturation

**Figure 1:**
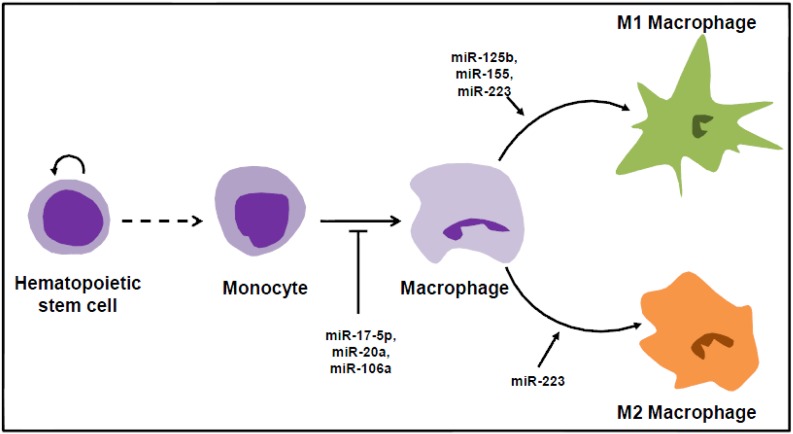
MicroRNA regulation of monocytic maturation and macrophage polarization.

miRNAs play crucial regulatory roles in modulating hematopoietic lineage determination and formation, including monocytopoiesis (Figure 1) [11,12,13]. miR-17-5p, miR-20a and miR-106a are reported to enhance blast proliferation and suppress monocytic differentiation. The combination of these three microRNAs suppresses acute myeloid leukaemia-1 (AML-1) protein and consequently up-regulates the macrophage colony-stimulating factor (M-CSF) receptor [14], suggesting a potential strategy of treating AML by inducing leukemic cell differentiation after transduction of miRNAs. In addition, several miRNAs, including miR-155, miR-222, miR-424 and miR-503, are downregulated in rheumatoid arthritis, and the restoration of these miRNAs leads to cell cycle arrest accompanied by monocytic differentiation in THP-1 cells [15]. This evidence strengthens the idea of using miRNA combinations to treat acute myeloid leukemia by the restoration of cell differentiation and the suppression of the proliferation of cancerous cells.

## 3. miRNA-Regulated Macrophage Infiltration

**Figure 2:**
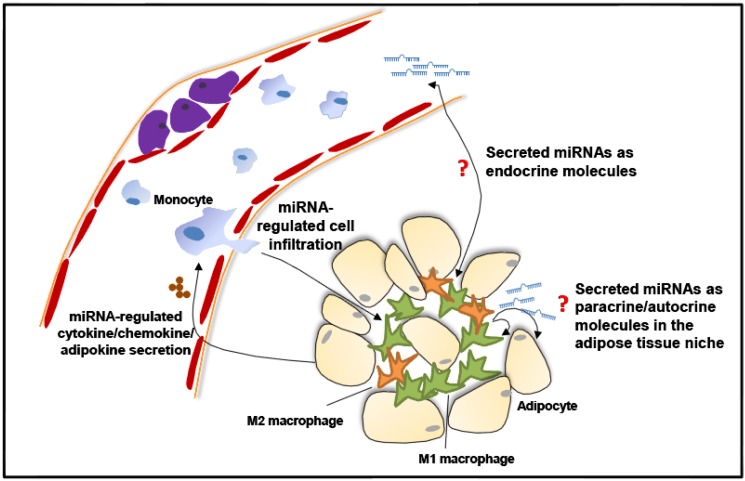
Action models of microRNA regulation in adipose tissue macrophages and foam cells of an atherosclerotic plaque.

Macrophage infiltration occurs in many tissue types, such as adipose tissue, the vascular system, liver and muscle. Circulating monocytes can sense the attractants released by certain cells, including endothelial cells and resident macrophages. CCL2 secretion accompanied by several adhesion molecules presenting on the cell surface can facilitate monocyte infiltration [5,6,16]. miRNAs can also affect monocyte infiltration via several mechanisms (Figure 2). For instance, miR-124a modulates CCL2 expression to facilitate the rolling and attachment of monocytes to the vessel wall [17]. Moreover, miR-17, miR-20a and miR-106a are involved in macrophage infiltration through the direct suppression of expression of signal-regulatory protein α (SIRP α) [18]. In addition, vascular smooth muscle cells that express high levels of miR-145 can decrease macrophage infiltration and, thus, could be targeted to develop a new therapeutic strategy to alleviate atherosclerosis [19]. miR-223 controls the infiltration of myeloid cells through directly targeting chemoattractants, such as chemokine C-X-C motif ligand 2 (CXCL2) and CCL3 [20]. In addition, our recent study revealed that depletion of miR-223 results in the elevated infiltration of macrophages into visceral adipose tissues of obese mice [21]. Interestingly, the inflammatory macrophages and the macrophages in atherosclerotic lesions exhibit an increase in the level of the expression of miR-155, subsequently suppressing the expression of B-cell CLL/lymphoma 6 (BCL6). The release from the direct repression of BCL6 allows CCL2 expression to increase in those macrophages [22,23]. Monocyte infiltration is a complex action involving multiple steps. Further investigation of the potential mechanisms underlying miRNA action in controlling monocyte infiltration is yet to be conducted.

## 4. miRNA-Regulated Macrophage Polarization

Strikingly, adipose tissue macrophages (ATMs) undergo a phenotypic switch from anti-inflammatory status (M2) in adipose tissues of lean individuals to a proinflammatory (M1) status in adipose tissues of obese subjects, which results in the development of tissue inflammation and systemic insulin resistance [24,25,26,27]. This activation pattern of macrophages is termed macrophage polarization, and it covers a full spectrum of activation stages between well-defined classical and alternative activation statuses. In brief, the M1 macrophages display classic proinflammatory responses depending on toll-like receptors (TLRs) and the activation of nuclear factor κB (NFκB)/c-Jun N-terminal kinase (JNK) signaling pathways. This cell signaling cascade leads to the release of a set of proinflammatory cytokines, such as tumor necrosis factor α (TNFα) and interleukin 1β (IL1β), which directly impair the insulin signaling pathway and activate the proinflammatory responses of adipocytes through paracrine mechanisms. In contrast, M2 macrophage activation leads to the production of a group of anti-inflammatory cytokines, such as IL4 and IL10, through the recruitment of peroxisome proliferator-activated receptor γ (PPARγ) or other transcription factors [28,29,30]. It has been demonstrated that M2 macrophage activation can improve systemic insulin sensitivity and protect against the development of cardiovascular diseases and type 2 diabetes [31,32,33]. Our recent findings revealed that in addition to the regulatory effects of cytokines, miRNAs can exhibit the profound functions on regulating macrophage polarization (Figure 1). miR-223 is differentially expressed during the activation of macrophage polarization, resulting in a high abundance in M2 macrophages [21]. Depletion of miR-223 significantly promotes macrophage infiltration and M1 macrophage activation, which subsequently elevates the state of inflammation in adipose tissues and exacerbates insulin resistance in the diet-induced obese mouse model. The increase in the expression of miR-125b, which can downregulate the expression of interferon regulatory factor 4 (IRF4) in macrophages, promotes the expression of cell surface marker CD80 in response to activation by interferon gamma (IFNγ) or following the activation of T-cells [34]. On the other hand, miR-155 is reported to respond to inflammatory cues during macrophage activation, which requires the activation of the JNK pathway [35]. Furthermore, JNK inhibitors block the induction of miR-155 in the inflammatory responses to macrophages [35]. Understanding the miRNA profiles associated with different pathways for macrophage activation could provide a new spectrum of markers for their identification and also inform the therapeutic approach to mitigate entry into tissues and the onset of macrophage-related diseases.

## 5. miRNA-Regulated Foam Cell Formation

Macrophage malfunction, including foam cell formation, is a critical problem in many physiological conditions. miR-155 has been shown to increase in macrophages after exposingto oxidized low-density lipoproteins (oxLDLs) in combination with IFNγ [35,36]. In addition, as the target of miR-155, HMG box-transcription protein1 (HBP1)-knockdown macrophages increase lipid uptake and reactive oxygen species (ROS) production after oxLDLs treatment [37]. Furthermore, miR-342-5p and miR-155 are upregulated in atherosclerotic lesions in ApoE(-/-) mice. By targeting the 3'-untranslated region of Akt1, miR-342-5p induces proinflammatory factors, nitric oxide synthase 2 (NOS2) and IL6 in macrophages, while the antagomir of miR-342-5p accelerates Akt1 expression [38]. These studies investigated the profile of miRNAs in oxLDLs-induced foam cells to provide a therapeutic strategy to manipulate miRNAs that relieve foam cell-induced cardiovascular syndrome (Figure 2). The infiltrated macrophages have generated significant research efforts, as they are believed to be the source of molecules that provide a critical switch of macrophage function and their regulation of the local environment in tissues.

## 6. miRNAs Secreted by Resident Macrophages

miRNAs expressed by macrophages not only regulate the biological function of macrophages *per se*, but they also exert regulatory effects on adjacent and remote cells or tissues through endocrine or paracrine signaling (Figure 2). In addition to intracellular regulation, the recent discovery of extracellular miRNAs provides another potential mechanism whereby miRNAs affect macrophage functions. Comparing plasma miRNA profiles from various diseases with normal controls, unique miRNA signatures were identified that displayed a high correlation with disease progression and prognosis, thus providing a new set of biomarkers for clinical diagnosis [39,40]. For instance, as a potential biomarker for cardiovascular diseases, there is an increase in the miR-1 level in patients with acute myocardial infarction (AMI) as compared with those without AMI [41]. In patients with type 2 diabetes, the expression of several circulating miRNAs, including miR-20b, miR-21, miR-24, miR-15a, miR-126, miR-191, miR-223, miR-320 and miR-486, are deceased compared to the healthy control group [41]. Moreover, miR-138, miR-15b and miR-376a can serve as predictive biomarkers in people with obesity [42,43]. To date, extracellular miRNAs have been reported to exist in several secretory formats, including exosomal particles, microvesicles and argonaute protein-associated complexes [44,45,46,47,48]. Of note, the expression profiles of miRNAs display distinct patterns between intracellular and exosomal compartments, suggesting that the secretion of miRNAs is well regulated and that they are packaged with certain proteins and structures to facilitate their action. However, our understanding of the secretion of extracellular miRNAs is still in its infancy, which greatly hinders their potential use as biomarkers or effective targets for drug development. The other major question regarding extracellular miRNA action to be clarified is how recipient cells respond to and selectively uptake extracellular miRNAs. At this time, two mechanisms have been proposed: exosome- or non-exosome-mediated transfer of miRNAs [49]. In addition, ligand-receptor interaction is a potential alternative strategy in target cells, for the microvesicles could be internalized by membrane fusion or endocytosis [50,51,52]. Thus, the extracellular miRNAs secreted by resident macrophages may function as potential endocrine/paracrine/autocrine molecules, which require their detailed mechanisms of action being investigated in future studies. 

## 7. Conclusion

It is clear that miRNAs, like other protein coding genes, can serve as biomarkers for diagnostic reference. The combination of signature miRNAs and protein factors can accelerate the accuracy of early diagnosis, so that proper treatments can be provided to benefit patients. On the other hand, although emerging evidence supports the critical roles of miRNAs in the development of atherosclerosis and obesity-associated metabolic disorders, the application of miRNAs as therapeutic targets needs to be further explored. Muthiah *et al.* reported that miR-145 can be delivered as an efficient therapeutic gene for atherosclerosis, resulting in the down-regulation of the target proteinsand the subsequent reduction in the proliferation of vascular smooth muscle cells [53]. A recent study in non-human primates showed that the inhibition of miR-33a/b has profound effects on increasing circulating HDL levels, but reduces VLDL triglycerides, suggesting the potential therapeutic application of antagomir in treating atherosclerosis [54]. However, given the regulatory position of miRNAs in the regulatory networks, altering one miRNA could suppress multiple target genes and the subsequent signaling pathways in which they are involved. In addition, the available target genes of a given miRNA can vary in different cells and disease contexts; an off-target outcome should also be considered. Longer term outcome analysis and potential interference monitoring in physiological systems other than targeted pathways and tissues are necessary to ensure treatment efficacy and safety in miRNA-related therapy. 

The resident macrophages in tissues and organs play critical roles in controlling physiological functions and systemic homeostasis in the host tissues. miRNAs are a set of potent regulators of macrophage differentiation, infiltration, activation and cell-cell interactions. Moreover, extracellular miRNAs could serve as a novel means for communication among cells to regulate the function of cells within the host tissues or in the remote target cells. To better understand the pathogenesis of macrophage-dependent diseases, including obesity-associated metabolic syndrome and cardiovascular diseases, further efforts are required to fully dissect the regulatory networks underlying the actions of miRNAs in controlling resident macrophage formation, infiltration and activation. Eventually, understanding the functions of miRNAs in macrophages will open a window to the development of novel therapeutic strategies to mitigate obesity-associated diseases and atherosclerosis. 
